# Biomarkers of Whale Shark Health: A Metabolomic Approach

**DOI:** 10.1371/journal.pone.0049379

**Published:** 2012-11-15

**Authors:** Alistair D. M. Dove, Johannes Leisen, Manshui Zhou, Jonathan J. Byrne, Krista Lim-Hing, Harry D. Webb, Leslie Gelbaum, Mark R. Viant, Julia Kubanek, Facundo M. Fernández

**Affiliations:** 1 Georgia Aquarium Research Center, Georgia Aquarium, Atlanta, Georgia, United States of America; 2 School of Chemistry and Biochemistry, Georgia Institute of Technology, Atlanta, Georgia, United States of America; 3 NERC Biomolecular Analysis Facility – Metabolomics Node (NBAF-B), School of Biosciences, University of Birmingham, Birmingham, United Kingdom; 4 School of Biology, Georgia Institute of Technology, Atlanta, Georgia, United States of America; Mayo Clinic, United States of America

## Abstract

In a search for biomarkers of health in whale sharks and as exploration of metabolomics as a modern tool for understanding animal physiology, the metabolite composition of serum in six whale sharks (*Rhincodon typus*) from an aquarium collection was explored using ^1^H nuclear magnetic resonance (NMR) spectroscopy and direct analysis in real time (DART) mass spectrometry (MS). Principal components analysis (PCA) of spectral data showed that individual animals could be resolved based on the metabolite composition of their serum and that two unhealthy individuals could be discriminated from the remaining healthy animals. The major difference between healthy and unhealthy individuals was the concentration of homarine, here reported for the first time in an elasmobranch, which was present at substantially lower concentrations in unhealthy whale sharks, suggesting that this metabolite may be a useful biomarker of health status in this species. The function(s) of homarine in sharks remain uncertain but it likely plays a significant role as an osmolyte. The presence of trimethylamine oxide (TMAO), another well-known protective osmolyte of elasmobranchs, at 0.1–0.3 mol L^−1^ was also confirmed using both NMR and MS. Twenty-three additional potential biomarkers were identified based on significant differences in the frequency of their occurrence between samples from healthy and unhealthy animals, as detected by DART MS. Overall, NMR and MS provided complementary data that showed that metabolomics is a useful approach for biomarker prospecting in poorly studied species like elasmobranchs.

## Introduction

Whale sharks, *Rhincodon typus* Smith 1828, are circumtropical planktivorous sharks and the largest fish in the world’s oceans [1], [2], [3]. They spend their adult lives as solitary individuals migrating across the open ocean or congregating in areas of intense productivity such as coastal upwelling zones in the tropics [1], [4], where plankton densities are higher than in nutrient-limited tropical surface waters [5]. Despite their size and increasing importance as a target for ecotourism operations [2], remarkably little is known about the internal biology of this species [6].

Maintenance of a population of six whale sharks in a large public aquarium in Atlanta, USA has provided opportunities to gather new information about their biology [7]. Two animals in the collection died in 2007 after periods of 3 and 7 months of inappetance, during which they were provided with supportive nutrition and intensive veterinary care. While the onset of their illness coincided with a series of anti-parasitic treatments applied to the exhibit, none of the other 50 species in the collection was affected (including two female whale sharks), necropsy findings were inconclusive and the ultimate cause of death for the two male animals remains unknown. Traditional serum chemistry indices obtained during veterinary examinations of these two animals did not correlate well with clinical observations [6]. Blood samples from these unhealthy individuals (hereafter referred to as Animals 1 and 2) provided data for comparative analyses with samples taken from three of the remaining four normal animals (hereafter Animals 3–6).

Due to the logistical challenges of working with such large animals rarely kept in captivity, blood samples had not been collected from captive or free-ranging whale sharks prior to this study. This material therefore presented a unique opportunity to research better biomarkers of health in this and other elasmobranch species. Published studies describing biomarkers in elasmobranchs are relatively few in number and have focused on enzymatic indices (see [8], [9], [10], [11], [12] for recent examples) but none have ever examined whale sharks, nor evaluated in detail the potential indicators of health among metabolites such as amino acids, sugars, fatty acids or non-peptide hormones.

Prompted in part by the unique nature of the samples, we used discovery-based metabolomic methods to provide the maximum amount of data without *a priori* knowledge of the composition of the samples: proton nuclear magnetic resonance (NMR) spectroscopy and direct analysis in real time (DART) mass spectrometry (MS) [13]. Metabolomics is the study of the low molecular weight (i.e. <1 kDa) molecules in a biological sample using bioanalytical and bioinformatic tools [14]. This approach has been reinvigorated recently by new technologies, allowing its application to understand metabolic perturbations such as those occurring during disease and exposure to toxicants [14], [15], [16], [17], [18]. In metabolomic studies, the progression of a disease can be observed as a trajectory deviating away from a “normal” state in principal component space [19].

Using NMR and MS metabolomic approaches, we sought to characterize variations in the metabolism of healthy and unhealthy whale sharks over a period of several months and thereby identify biomarkers of health in this elasmobranch species. We succeeded in distinguishing healthy and unhealthy animals and identified several promising biomarker compounds.

## Results

### 1. Metabolic Profile of the Whale Shark

**Figure 1:**
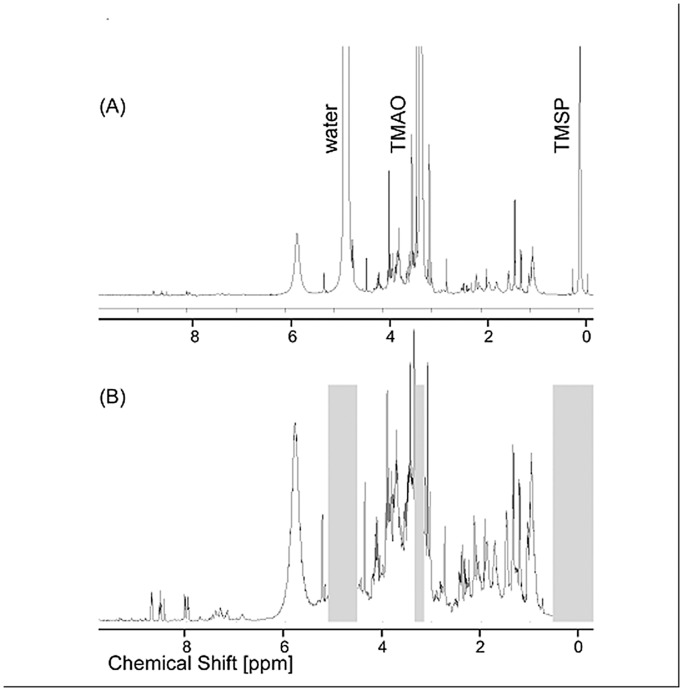
Typical ^1^H NMR spectrum of whale shark serum: (a) Full spectrum: the signal at 3.27 ppm corresponds to trimethylamine-N-oxide (TMAO). Two other major peaks are due to an internal reference (TMSP; used for referencing the chemical shift scale) and residual protons in the solvent. (b) Spectrum (a) after pre-processing (see Methodology). The grey bars depict spectral regions which were excluded from PCA.

NMR and MS analyses of serum samples in this study represent the first examination of the physiology of the world’s largest fish. The ^1^H NMR spectra of serum extracts revealed the presence of a complex mixture of chemical species in 46 samples collected over a period of months from five whale sharks (Fig. 1A). Consistent with most vertebrate metabolism, the serum of whale sharks was dominated by amino acids involved in protein synthesis and hydroxy-acids involved in energy metabolism (Table 1). Yet there are some notable differences from other vertebrate groups. Trimethylamine oxide (TMAO), for example, was abundant in healthy whale shark serum samples (Fig. 1). It is a well-known osmolyte in sharks and other marine species, but is not present in appreciable quantities in mammals [20], [21]. Similarly, intermediaries in the urea cycle were prominent in the metabolic profiles of whale sharks, which is perhaps not surprising given the important role of urea in the osmotic homeostasis of this and all shark species. Even more striking, homarine (*N*-methyl picolinic acid) is here reported for the first time from any elasmobranch species (see Appendix).

### 2. Metabolomic Analyses Distinguish Healthy from Unhealthy Whale Sharks

**Figure 2:**
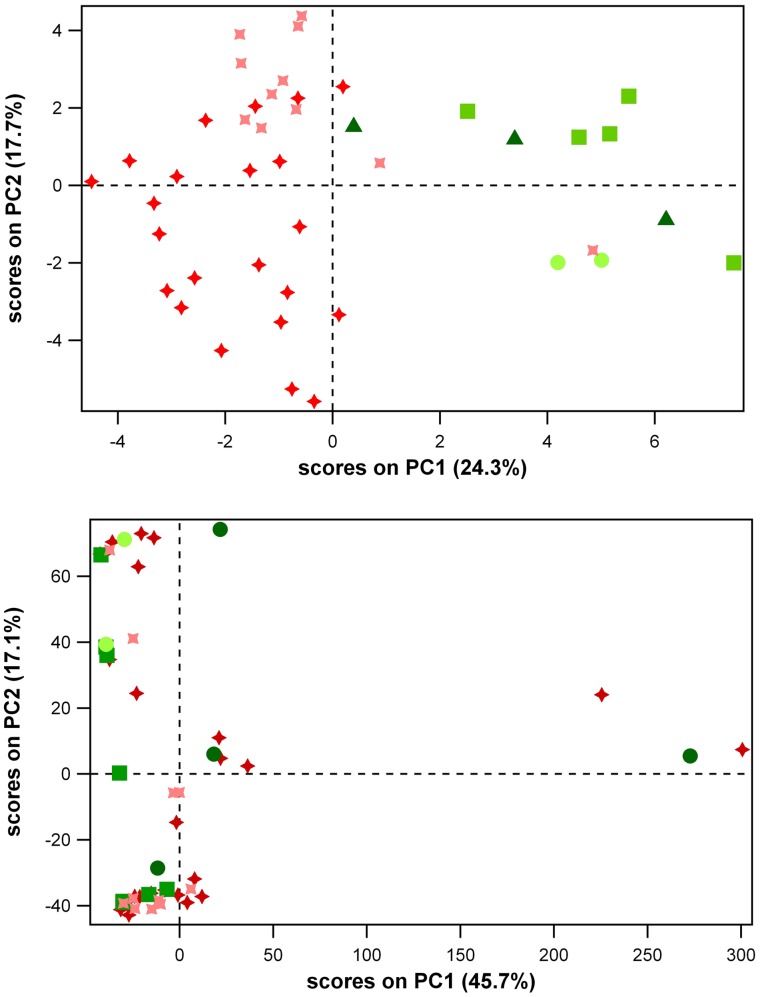
PCA scores plots from analysis of (a) NMR and (b) MS metabolomics datasets of only those whale shark serum samples that were analysed by both methods. 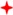
: unhealthy individual 1 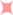: unhealthy individual 2 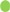: healthy individual 3 (n = 2) 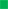: healthy individual 4 (n = 3) 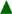: healthy individual 5 (n = 5).

**Figure 3:**
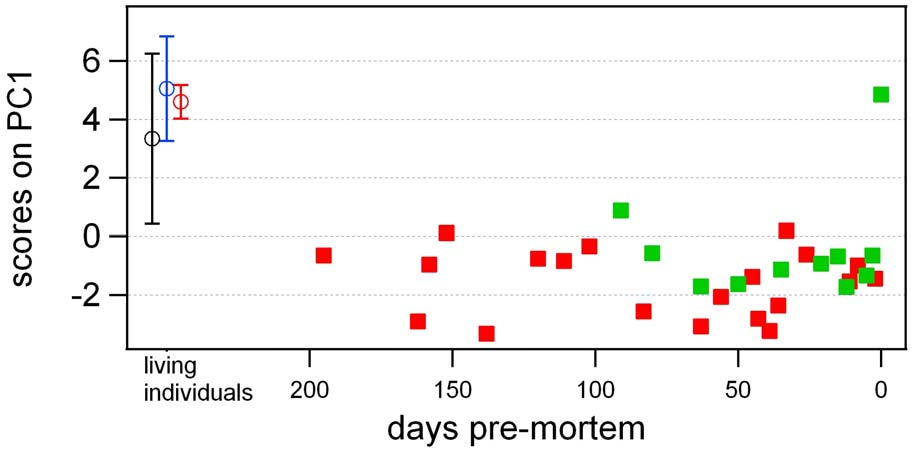
PCA of ^1^H NMR spectra of extracted whale shark serum (42 samples, showing PC1 scores plotted against time of sampling for the unhealthy animals. 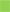
: unhealthy individual 1 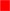: unhealthy individual 2 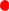: average for healthy individual 3 (n = 2) 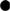: average for healthy individual 4 (n = 3) 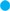: average for healthy individual 5 (n = 5).

Metabolic profiles of unhealthy whale sharks were significantly different than those of healthy individuals. Pre-processing of these spectra (Fig. 1B) to remove sampling artifacts and the overwhelming influence of the most abundant metabolite, TMAO, allowed statistical evaluation of NMR spectral data by principal component analysis (PCA). Distinct separation of serum samples from the two unhealthy (Animals 1–2) versus the three healthy individuals (Animals 3–5) was evident in the first two principal components (PC1 and PC2), which together accounted for 42% of the variance in the NMR dataset (Fig. 2A). Low or negative scores on the first component (PC1) alone allowed discrimination of almost all samples originating from unhealthy individuals, except on the last day of the life of Animal 1 when veterinary intervention (intravenous dextrose) altered the metabolic profile of this individual (Fig. 3).

**Figure 4:**
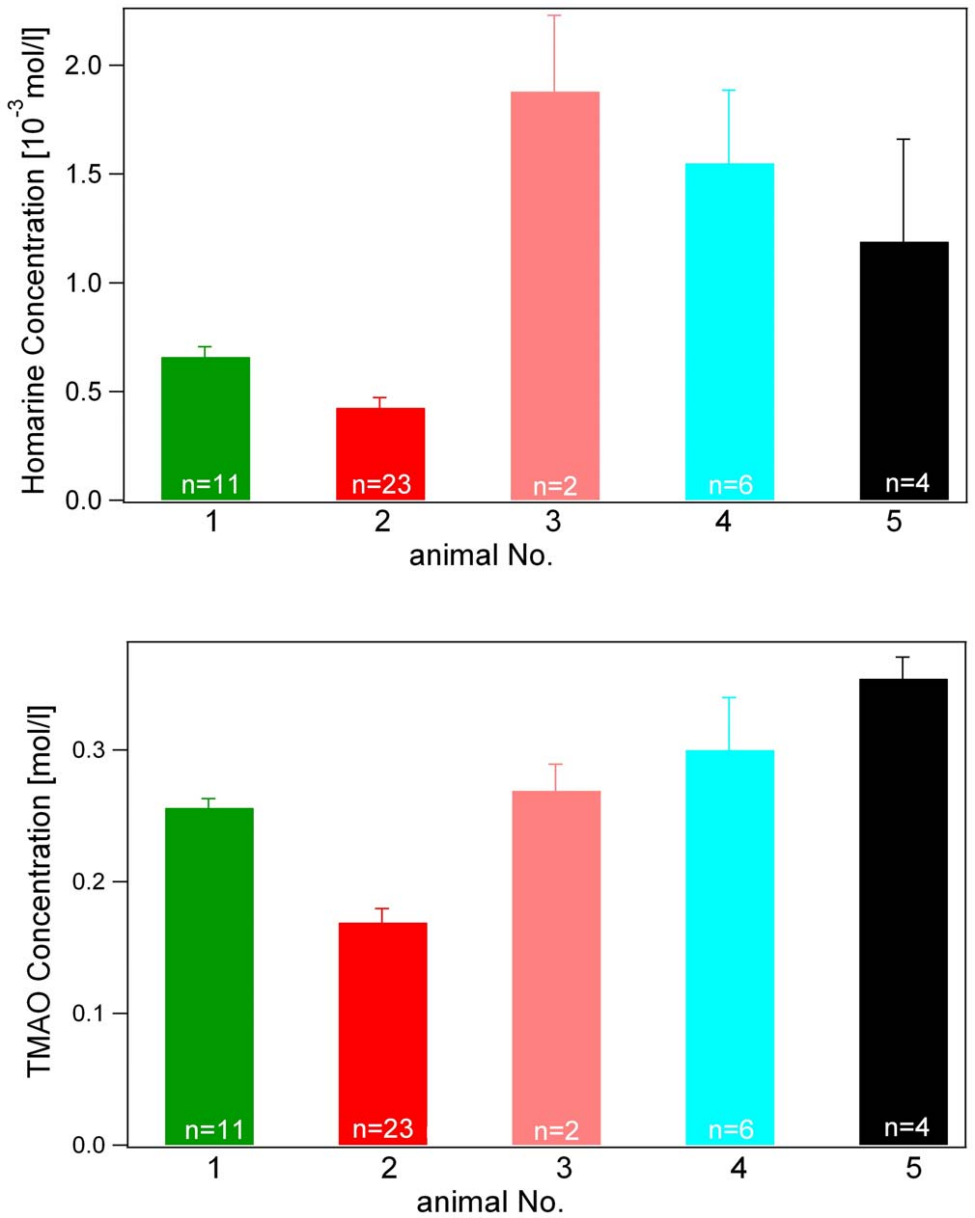
Differences in concentration of homarine (A) and trimethylamine-oxide (TMAO) (B) in serum samples from two unhealthy (animals 1–2) and three healthy (3–5) whale sharks.

PCA analysis of mass spectra from 53 serum samples from all six whale sharks did not distinguish individuals based upon frequency of occurrence of individual metabolites (Fig. 2B). However, MS analyses provided tentative identification of hundreds of metabolites, of which approximately 70 were present in at least half of all samples. The lists of more commonly detected candidate compounds were then subjected to frequency analyses to extract additional patterns from the data set.

### 3. Small Molecules as Biomarkers

**Figure 5:**
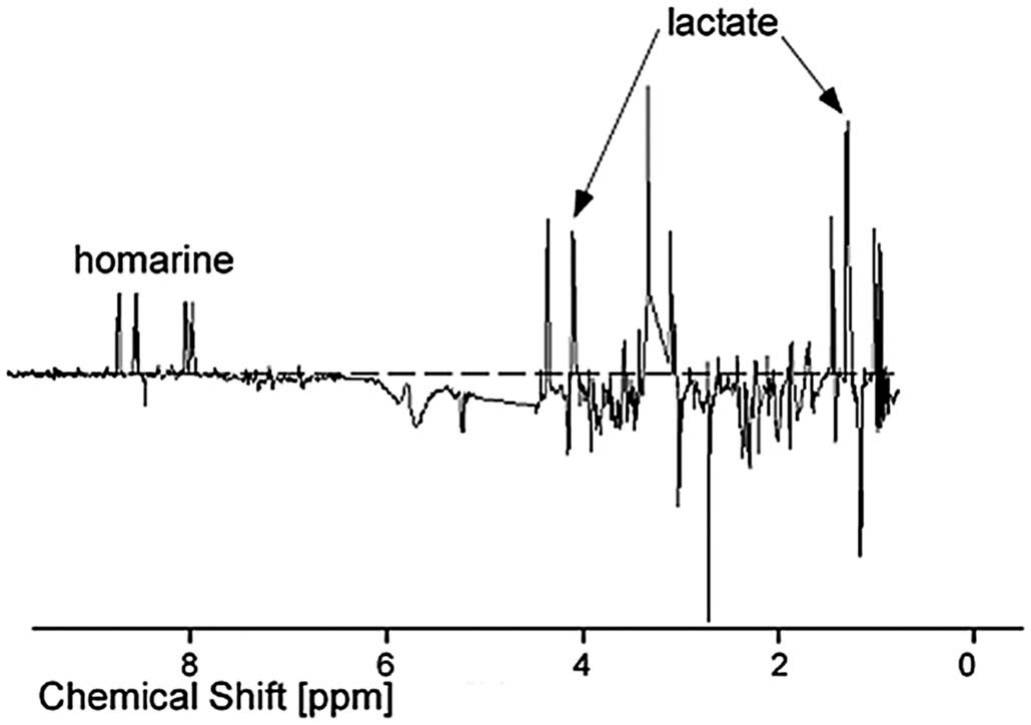
Loading plot for PC1 as a means to identify NMR spectroscopic features corresponding to relevant metabolites within the serum of whale sharks.

From ^1^H NMR spectral analysis, the heteroaromatic metabolite homarine was recognized as the component of whale shark serum contributing the greatest loading to PC1, which best separated healthy from unhealthy animals and was therefore considered a promising biomarker. The identity of this metabolite was first assigned to homarine (*N*-methyl picolinic acid) by liquid chromatography coupled to tandem MS and ^1^H NMR spectroscopy, and then confirmed by total synthesis and spectroscopic comparison of synthetic homarine and whale shark serum samples (see Appendix). Comparing peak areas of aromatic proton signals from 46 whale shark serum extracts with peak areas of an internal standard (deuterated trimethylsilylpropionate [TMSP]) of known concentration, we calculated that homarine was present in healthy whale shark serum at a concentration of approximately 1.5 mM, compared with 0.5 mM for unhealthy individuals (Fig. 4A; p<0.05 for each unhealthy animal vs. each healthy animal by ANOVA with Tukey post-hoc test; n = 2–23 samples for each individual). The concentration of homarine also declined somewhat for unhealthy Animal 1 during its time series, although this trend was not observed for the other unhealthy individual. In addition to homarine, lactate also contributed strongly to the loadings for PC1 (Fig. 5).

**Table 1:** “Core” candidate metabolites detected and tentatively identified by DART MS from whale shark serum samples*.

Tentative metabolite ID	P_H_	P_M_	Class	Function (mammalian)
**Trimethylamine oxide (TMAO)**	1.00	1.00	Aliphatic amine	Osmolyte (shown for elasmobranchs also)
**2-Ethyl-2-hydroxybutyric acid**	1.00	0.865	Short-chain hydroxy acid	Not a major mammalian metabolite
**2-Hydroxy-3-methylpentanoic acid**	1.00	0.919	Short-chain hydroxy acid	Metabolite of isoleucine
**2-Hydroxycaproic acid**	1.00	0.865	Short-chain hydroxy acid	Endogenous but normal function uncertain
**5-Hydroxyhexanoic acid**	1.00	0.865	Short-chain hydroxy acid	Omega-oxidation product of fatty acids
**D-Leucic acid**	1.00	0.865	Short-chain hydroxy acid	Endogenous but normal function uncertain
**Hydroxyisocaproic acid**	1.00	0.865	Short-chain hydroxy acid	Metabolite of branched chain amino acids
**Leucinic acid**	1.00	0.865	Short-chain hydroxy acid	Normal function uncertain, known bacterial metabolite
**N-Acetylglutamine**	1.00	0.811	Amino acid	Stable analogue of glutamine, protein synthesis
**Urea**	1.00	0.892	Amino-ketone	Osmolyte (elasmobranchs), protein catabolyte (mammals)
**Acetic acid**	0.938	0.865	Short-chain fatty acid	Metabolism of CoA, carbohydrates and fats
**Carbon Dioxide**	0.938	0.811	Gas	Respiratory end-product
**Glycolaldehyde**	0.938	0.865	Alcohol/aldehyde	Precursor of CoenzymeA
**3,4-Dihydroxyphenylglycol**	0.875	0.703	Alcohol/polyphenol	Norepinephrine metabolite
**Cinnamaldehyde**	0.875	0.84	Short-chain aldehyde	Plant metabolite (possible misidentification)
**Dihydropteridine**	0.875	0.649	Heterocyclic amine	Component of folate synthesis
**Dimethylsulfide**	0.875	0.676	Gas	Osmolyte, enzyme cofactor, signaling molecule
**Trigonelline**	0.875	0.730	Amino acid	Exogenous in mammals
**1-deoxy-D-xylulose**	0.813	0.622	Monosaccharide	Metabolite of pyridoxine, involved in vitamin B6 metabolism
**2,3-Dihydroxyvaleric acid**	0.813	0.622	Short-chain hydroxy acid	Endogenous but normal function uncertain
**Deoxyribose**	0.813	0.622	Monosaccharide	DNA architecture, energy metabolism (via role in ATP)
**Imidazole**	0.813	0.649	Heterocyclic amine	Component of many biological molecules
**2-Methylacetoacetic acid**	0.750	0.838	Short-chain keto-acid	Intermediate in synthesis and degradation of ketones
**2-Oxovaleric acid**	0.750	0.784	Keto/fatty acid	Valine, leucine and isoleucine metabolite
**a-Ketoisovaleric acid**	0.750	0.784	Short-chain keto-acid	Precursor in leucine and valine synthesis
**Levulinic acid**	0.750	0.784	Short-chain keto-acid	Component of porphyrin and chlorophyll metabolism
**Methylacetoacetic acid**	0.750	0.784	Short-chain keto-acid	Endogenous but normal function uncertain

* P_H_ and P_M_ refer to the proportion of total healthy (n = 16) and unhealthy (n = 37) shark samples, respectively, from which each compound was identified.

**Table 2:** Candidate biomarker metabolites detected and tentatively identified by DART MS that showed a significant difference in frequency between healthy (H) and unhealthy (M) whale sharks (two-proportion z-test *p*<0.05) after Bonferroni correction.

Tentative metabolite ID	P_H_*	P_M_^†^	*z*	*p*	Corr. *p*	Class	Function (mammalian)
Saccharopine	0.625	0.189	3.12	0.001	**0.021**	Amino acid	Principal normal metabolite of lysine catabolism
L-Asparagine	0.688	0.324	2.46	0.007	0.164	Amino acid	Essential amino acid
Ureidopropionic acid	0.688	0.324	2.46	0.007	0.164	Amino acid	Urea cycle; CoA, pyrimidine & alanine metabolism
D-Ornithine	0.688	0.351	2.26	0.012	0.275	Amino acid	Urea cycle; arginine & proline metabolism
Ornithine	0.688	0.351	2.26	0.012	0.275	Amino acid	Urea cycle; component of several amino acid metabolisms
Pantetheine	0.688	0.378	2.07	0.019	0.440	Tripeptide	Intermediate in vitamin B and CoA metabolism
N-Acetylglutamine	1.000	0.811	1.87	0.031	0.713	Amino acid	Amino acid metabolism, especially glutamine
Dihydropteridine	0.875	0.649	1.68	0.047	1.076	Heterocyclic amine	Folate biosynthesis
Carbamic acid	0.625	0.378	1.66	0.048	1.105	Amino acid	Protein synthesis, amino acid biosynthesis
Heptanoic acid	0.625	0.378	1.66	0.048	1.105	Carboxylic acid	
*Diacetyl*	*0.313*	*0.622*	*−2.07*	*0.019*	*0.446*	*Ketone*	*Product of malolactic fermentation*
*gamma-Butyrolactone*	*0.313*	*0.622*	*−2.07*	*0.019*	*0.446*	*Short chain FA*	* 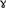 -aminobutyric acid catabolite*
*Oxolan-3-one*	*0.313*	*0.622*	*−2.07*	*0.019*	*0.446*	*Ketone*	*Urinary marker found in lactic acidosis*
*2-Ketohexanoic acid*	*0.375*	*0.703*	*−2.24*	*0.013*	*0.289*	*Keto acid*	*Inhibits insulin homeostasis*
*2-Methyl-3-ketovaleric acid*	*0.375*	*0.703*	*−2.24*	*0.013*	*0.289*	*Keto/Hydroxy acid*	*Leucine catabolite in keto-acidosis*
*3-Methyl-2-oxovaleric acid*	*0.375*	*0.703*	*−2.24*	*0.013*	*0.289*	*Keto acid*	*Isoleucine catabolite*
*Ketoleucine*	*0.375*	*0.703*	*−2.24*	*0.013*	*0.289*	*Keto acid*	*Neurotoxic amino acid catabolite*
*4-Hydroxycyclohexylacetic acid*	*0.188*	*0.622*	*−2.90*	*0.002*	***0.043***	*Hydroxy acid*	*Dysfunctional tyrosine metabolite*
*2-Methylglutaconic acid*	*0.125*	*0.568*	*−2.98*	*0.001*	***0.033***	*Dicarboxylic acid*	*Product of metabolic acidosis found in aciduria*
*3-Hexenedioic acid*	*0.125*	*0.568*	*−2.98*	*0.001*	***0.033***	*Dicarboxylic acid*	*FA metabolite found in aciduria*
*3-Methylglutaconic acid*	*0.125*	*0.568*	*−2.98*	*0.001*	***0.033***	*Dicarboxylic acid*	*Catabolic leucine metabolite found in aciduria*

Unlike homarine and lactate, the variance in TMAO concentration did not distinguish all healthy from all unhealthy whale shark serum samples (Fig. 4B). Nevertheless, Animal 2 exhibited approximately 50% lower concentration of this important osmolyte relative to healthy Animals 4–5 whose serum contained 0.30–0.35 M TMAO, a difference that was found to be statistically significant by ANOVA followed by Tukey post-hoc analysis. Given that serum concentrations of TMAO were not significantly different for unhealthy Animal 1 vs. two of the healthy animals, it does not appear that TMAO is a reliable biomarker indicating whale shark health. Urea, another well-known osmolyte contributing up to 300 mOsm to shark serum, was not directly detected by ^1^H NMR spectroscopy due to chemical exchange of its protons with the deuterons in the solvent and so could not be considered for biomarker potential in this study.

Hundreds of metabolites were tentatively identified by their DART mass spectral data using the publically-available metabolite database METLIN (http://metlin.scripps.edu/). In contrast, matching of NMR spectra with publically-available data using an online search engine (Madison, BMRB) [22] returned few exact matches, possibly due to a combination of novel aspects of the elasmobranch metabolome and spectroscopic challenges including overlap of ^1^H NMR signals in whale shark serum samples. While the DART MS approach in its present implementation did not provide quantitative data related to concentration of each analyte, the analysis of presence vs. absence of compounds by MS was highly informative because of the better sensitivity and resolving power of MS compared to NMR, and because many metabolites appeared to occur at concentrations near or below the MS detection limit, such that they were apparently “absent” from some samples while detectable in others.

Overall, twenty-seven compounds including TMAO and urea were detected in at least 70% of all samples analyzed by DART MS from healthy and unhealthy individuals (Table 1). Homarine was detected by DART in 22% of the samples, but because of the potential for unwanted fragmentation during DART ionization of this type of labile N-substituted species [23], DART homarine signals were not used in our frequency analysis. Twenty-three additional compounds occurred in significantly different proportions of samples for healthy vs. unhealthy animals (Table 2). Decreased frequencies of ten of these biomarkers in serum samples of unhealthy individuals indicate apparent deficiencies in urea cycle, amino acid biosynthesis and catabolism, vitamin metabolism, and folate biosynthesis. Unhealthy sharks exhibited increased frequencies of 13 biomarkers that, in mammals, typically correlate with acidosis, aciduria, dysfunctional amino acid metabolism, and other indicators of abnormal metabolism (HMDB [24]) (Table 2). After Bonferroni correction for multiple comparisons, five metabolites remained significantly different between healthy and unhealthy animals; saccharopine was more frequently detected in healthy whale sharks, while 4–hydroxycyclohexylacetic acid, 2-methylglutaconic acid, 3-hexenedioic acid and 3-methylglutaconic acid were all more frequently detected in unhealthy animals.

## Materials and Methods

### 1. General

Blood samples were collected from six whale sharks from 2006 through 2008. Animals 1 and 2 were classed as unhealthy and were sampled from October 2006 to June 2007. Animals 3, 4, 5 and 6 were classed as healthy and were sampled haphazardly from 2007 to 2008. Animals 1, 2, 5, and 6 were male. The age of the animals was unknown at the time of collection but estimated to be between 5 and 8 years for all animals in the study. Veterinary exams were conducted as described by Dove et al [6]. Briefly, individual animals were corralled by SCUBA divers into a vinyl stretcher suspended in their exhibit and sedated with hyperoxic water (120–150% saturation at 25°C) delivered towards the mouth with a flexible hose attached to a jacuzzi pump. Blood samples were then collected from the ventral caudal vein using a syringe connected to a 3.5 inch spinal needle by a 15 inch extension set and then allowed to clot in plain serum tubes (Becton Dickinson Co., Franklin Lakes NJ, USA), before being centrifuged for 10 min at 3,500 rpm (Eppendorf compact centrifuge, Hamburg, Germany) to separate the clot from the serum. Serum was drawn off in 2.0 mL aliquots, placed in CryoPro® cryovials (VWR, Westchester PA, USA) and frozen at −80°C for later analysis.

### 2. Sample Preparation

Whale shark serum (250 µL from each sample) was transferred to a clean 2.0 mL Eppendorf tube on ice and 500 µL of ice-cold acetonitrile was added to precipitate proteins. The tube was immediately sealed to prevent evaporation and the sample vortexed for 15 seconds and then centrifuged for 5 min at 15,000 rpm. The supernatant was then transferred to a clean cryovial tube, and then lyophilized.

For NMR studies the samples were re-suspended in 475 µL of deuterium oxide containing 20 mM of 3-trimethylsilyl–2,2,3,3-d4-propionate (TMSP), an internal standard with respect to the resonance frequency and concentration of metabolites. For MS studies the samples were re-suspended in ultrapure water and derivatized following our previously published protocol [13].

### 3. NMR Metabolomics Studies

A total of 46 samples from five whale sharks (Animals 1–5) were investigated by ^1^H NMR spectroscopy. NMR spectra were recorded on a Bruker-Biospin AMX400 spectrometer (Bruker, Germany) operating at 400.13 MHz equipped with a 5 mm broadband probe. For each spectrum 128 scans with a repetition time of 5 s, a spectral width of 20.8 ppm and 64K data points were accumulated using 30 degree excitation pulses. The scale of the chemical shift was calibrated with respect to TMSP (0.00 ppm).

NMR spectral data were analyzed using the MATLAB Bioinformatics Toolbox (MathWorks Inc., Natick MA, USA). Preporcessing steps required prior to Principal Component Analysis (PCA) were performed using the MATLAB script ProMetab (preliminary distribution of the version ProMetab GUI) [18]. These steps included: selective of a relevant spectral range (0–9 ppm), exclusion of undesirable spectral ranges (residual water at 4.5–5.1 ppm, TMAO at 3.24–3.3 ppm, TMSP at 0–0.5 ppm), data binning in steps of 0.005 ppm, baseline correction, normalization of spectra with respect to the the total spectra area (TSA), and glog transformation. PCA was then performed using PLS toolbox 5.2.2 (Eigenvector Research Inc., Wenatchee WA, USA). Data were mean-centered prior to the analysis. Results were plotted using IGOR Pro (WaveMetrics Inc., Lake Oswego OR, USA).

Based on the trends observed in the PCA, concentrations of the regions in the NMR spectra corresponding to homarine were compared between whale sharks by ANOVA. The concentrations were extrapolated using the chemical standard and then compared amongst the different whale sharks. Pair-wise comparisons between the concentrations were made using a Tukey-Kramer HSD post-hoc test.

### 4. MS Metabolomics Studies

A total of 53 samples from all six whale sharks were investigated by mass spectrometry. MS metabolomics analysis was performed via a DART ion source (IonSense, Saugus MA, USA) coupled to an AccuTOF mass spectrometer (JEOL, Tokyo, Japan) as previously described [13]. The DART ion source was operated in positive ion mode with a helium gas flow rate of 3.0 Lmin^−1^ heated to 200°C. Accurate mass spectra were acquired within the range of *m/z* 60–1000 with a spectral recording interval of 1.5 s. Mass drift compensation was performed after analysis of every sample using a 0.20 mM PEG 600 standard in methanol. Prior to PCA, mass spectra were normalized to the base peak intensity in Excel 2003 (Microsoft Corporation, Redmond, WA), imported as csv files, and resampled to 20,000 *m/z* points between 60 and 990 using the *msresample* function in the MATLAB Bioinformatics Toolbox.

## Discussion

Metabolomic approaches revealed multivariate data patterns of serum composition that paralleled observed differences in health status of individual whale sharks, indicating that declining health in this species can be recognized by blood chemical (metabolite) profiles (Fig. 2–3). Analysis by NMR and MS led to the identification of several potential biomarkers; that is, individual compounds that vary with health status in a seemingly meaningful way. These two outcomes confirm that metabolomic methods are useful tools for studying the health of aquatic animals, consistent with previous studies [14]. Overall, metabolomics produced a tremendous data return, thus maximizing the benefit that could be extracted from samples that are so difficult to gather and which have not yet been achieved in natural environments.

After preliminary data processing, PCA of ^1^H NMR spectra of whale shark serum extracts showed substantial differences between healthy and unhealthy whale sharks in the overall composition and concentration of metabolites in serum (Figs. 2–3). Unhealthy animals grouped together on PC1, a clustering pattern that was largely driven by fluctuations in the aromatic region of the spectrum, which was subsequently shown to be primarily due to the influence of homarine (Fig. 5; Appendix S1). Univariate analysis of homarine measured from individual samples by NMR confirmed that differences in homarine concentration between unhealthy and healthy whale sharks were statistically significant (Fig. 4A).

Although NMR spectroscopy was useful for characterizing metabolomes in unsupervised multivariate analyses, there was little congruence between lists of candidate compounds produced by our ^1^H NMR experiments and major online databases of metabolites, regardless of health status of the animal from which the sample was drawn. This may have been due to the complexity of the serum mixture, the inherent insensitivity of NMR, and the exchange of protons on some metabolites by deuterons from the solvent. Given that many metabolites common to eukaryotic organisms were identified from mass spectra of these same samples (Tables 1–2), it seems unlikely that whale shark metabolism differs fundamentally and substantially from model organisms studied previously. The MS dataset provided more information regarding individual compounds due to greater spectral resolution, but given our frequency-based approach of analyzing MS spectral data, was less useful for distinguishing healthy and unhealthy animals by PCA (Fig. 2B). Frequency analysis of the candidate compounds, however, provided another dimension of usefulness in the MS dataset and identified a number of promising biomarker molecules (Table 2). Overall, NMR and MS approaches were complementary towards the main goal of characterizing physiological indicators of ill health in this large, metabolically complex animal species.

We expected to detect trimethylamine oxide (TMAO), based on published studies reporting that high concentrations of this protective osmolyte are apparently universal in the blood of elasmobranchs (e.g., [25]). Concentrations of TMAO varied somewhat between animals such that the animal with the longest disease progression had the most reduced serum concentration of this metabolite, but this trend was not significant enough to discriminate all healthy from both unhealthy animals (Fig. 4B), suggesting that while it may serve important functions, this compound may not be a useful biomarker of health in whale sharks. It seems likely that, due to its critical role as an osmolyte that protects against the harmful effects of urea (also retained in shark blood at high concentrations), TMAO concentrations are both high and relatively constant.

In contrast to TMAO, homarine was determined to be a potentially useful biomarker because concentrations varied significantly between healthy and unhealthy animals (Fig. 4A). Homarine is widely distributed among marine taxa and is particularly abundant in invertebrate groups including sponges [26], gorgonians [27], corals [28], gastropods [29], bivalves [30], squids [31], holothurians [26], annelids [32], crustaceans [33], [34] and ascidians [35]; it even occurs in some phytoplankton species [36]. Among these groups it has been proposed to have a similarly great diversity of functions: an osmolyte [34]; a pattern control modulator during development [37]; an antifouling compound [38]; a predation deterrent [39]; an antibacterial agent [28] and an immune effector molecule [27]. Homarine has been less commonly reported from teleost fish, but does occur throughout the tissues of marine - but apparently not freshwater - fishes [40], indicating a probable osmolytic function among fishes. Since this is the first time that homarine has been reported from an elasmobranch, its specific functions in whale sharks are unclear. It may act as a protective osmolyte in a similar way as TMAO (albeit at much lower concentrations), or it may simply reflect dietary intake of the native compound or one of its precursors. Either hypothesis is supported by the lower concentrations of both homarine and urea cycle metabolites in unhealthy versus healthy sharks (Fig. 4A; Table 2). Why might homarine concentrations be lower in unhealthy whale sharks? Most likely, the anorexic nature of the illness in this case resulted in the animals not receiving necessary dietary sources of homarine or its precursors. A similar explanation may apply to the lower frequencies of precursors and products of amino acid, vitamin, and folate metabolism in unhealthy whale shark serum samples (Table 2).

In addition to uncertainty regarding the physiological significance of metabolites pinpointed due to their differential concentrations in healthy versus unhealthy whale sharks, the metabolism of whale sharks in general (e.g., involving metabolites identified in all samples, see Table 1) warrant further study. Specifically, an understanding of critical metabolites can improve conceptual models of elasmobranch metabolism, determine how it differs from teleosts and other vertebrates, and show how it supports the unique adaptations of whale sharks, such as the extraordinary deep diving described by Brunnschweiler et al. [41] and Graham et al. [42]. The logical next step would be to extend these approaches to samples collected from the field, whereby biomarkers identified in the aquarium setting could be used to assess health status in wild whale shark populations (“environmental metabolomics” *sensu* Hines et al. [19]). This is especially relevant wherever these populations are threatened by anthropogenic factors or environmental changes. Obviously there are tremendous logistical challenges inherent in that sort of study, but the potential research returns are great and could result in some much-overdue leaps forward in our understanding of this, the world’s largest fish species.

## Supporting Information

Appendix S1. (DOCX)

Appendix Figure S1 Identification of homarine in whale shark serum using LC-QTOF MS. (a). Total ion chromatogram (TIC) of a partially-purified whale shark serum sample. (b). Mass spectrum of the peak at 3.76 min in (a). Product ion QTOF MS/MS spectrum of the precursor ion at m/z 138 with collision energy of 20 eV from (c): partially-purified whale shark serum samples and (e): synthesized homarine. (d). Extracted ion chromatograms of ions at m/z 138 from whale shark serum samples (black curve) and synthesized homarine (blue curve). (f). Suggested fragmentation pathway for homarine.
(DOCX)
