# Supplementary material for: Biomarkers of Whale Shark Health: A Metabolomic Approach
Source: PLoS One. 2012 Nov 15;7(11):e49379. doi: 10.1371/journal.pone.0049379 (PMC3499553; doi:10.1371/journal.pone.0049379)
Supplement: Appendix S1. — (DOCX) [file pone.0049379.s001.docx]

**Supporting Information (Appendix)**

Characterization of homarine as a biomarker in whale sharks

*Homarine synthesis*

A mixture of ethyl picolinate (1.0 mL, 7.4 mmol, Sigma) in EtOH (5 mL) and iodomethane (1.36 mL, 21.8 mmol, Sigma) was heated at 100 °C for 12 hours. The mixture was then chromatographed on DOWEX 550A OH anion exchange resin (Sigma). The analyte was eluted with 8% aqueous NaCl and hydrolyzed with addition of aqueous NaOH solution to a final NaOH concentration of 0.01 M. The solution was then incubated at 80°C for 2 hours, and diluted with water prior to LC-MS analysis.

*Identification of homarine by LC-MS*

Homarine structural identification was performed by using HPLC combined with a QTOF mass spectrometer (Bruker micr-OTOF-Q I, Germany). 15 μL of shark serum sample was injected onto a reverse phase analytical C18 column (Symmetry^®^, 3.5 μm, 2.1 × 150 mm, pore size 100Å, Waters, Milford, MA) installed in an Agilent 1100 Series LC system (Santa Clara, CA). Positive ion mode ESI spectra were collected in the range of 50-400 *m/z*. The settings for the QTOF mass spectrometer were as follows: capillary voltage: ‑4500 V, end plate offset: -500 V, drying gas flow: 4.0 L min^-1^, nebulizer: 0.4 bar, drying gas temperature: 185ºC. The minimum measured resolving power of the QTOF mass spectrometer was 16,500 at FWHM for *m/z* 151.10, with observed mass accuracies in the range 0.6-2.0 ppm. The instrument was mass calibrated prior to performing experiments using a custom mixture of trimethylamine, PEG 400 and PEG 600 in methanol (1:2:2:1200 v/v), and all mass spectra were centroided in real time.

The total ion chromatogram (TIC) of partially-purified shark serum showed two peaks with retention time (RT) at 3.60 and 3.76 minutes, respectively (Appendix Fig. 1a). The short retention times indicated that these analytes corresponded to highly hydrophilic compounds. The compound with RT = 3.60 min was identified as TMAO by accurate mass measurements (data not shown). The mass spectrum corresponding to the TIC peak at 3.76 min showed a base peak at *m/z* 138.0558 ([M+H]^+^), and a secondary signal at *m/z* 160.0350 ([M+Na]^+^, Appendix Fig. 1b). Further investigation of the accurate mass spectrum (Appendix Fig. 1b) using a freely-distributed system of macros that relies on a series of heuristic rules to identify possible formulae based on the mass accuracy of the peak of interest and the corresponding isotopic ratios [[43](#_ENREF_43)] resulted in a candidate C_7_H_7_NO_2_ elemental composition with a score of 99%.

Appendix Fig. 1c shows the QTOF product ion MS/MS spectrum starting from the precursor ion at *m/z* 138.0558 (20 eV collision energy). The fragment ion at *m/z* 78.0288 was compatible with the pyridyl cation (C_5_H_4_N^+^). The accurate mass difference between various fragment ions was interpreted as follows : ∆m (138-106) = 32.0281 (M_W_ CH_3_OH = 32.0262), ∆m (138-124) = 14.0193 (M_W_ CH_2_ = 14.0156), ∆m (138-94) = 43.9933 (M_W_ CO_2_ = 43.9898) and ∆m (138-78) = 60.0270 (M_W_ C_2_H_4_O_2_ = 60.0270). This fragmentation pattern suggested the presence of a carboxylic acid group as substituent in a pyridine ring, which was consistent with the observed chromatographic retention times. Homarine, or one of its isomers, were chosen as potential identities for this compound matching the candidate elemental formula.

In order to confirm the identification of homarine, this compound was synthesized based on the method described by Polychronopoulos et al. [[44](#_ENREF_44)]. When the homarine extracted ion chromatogram in shark serum (black curve) was superimposed on the synthesized homarine (blue curve), their retention times differed only by 0.02 min, within the method variance (Appendix Fig. 1d). The product ion spectrum of the synthetic protonated homarine molecule ion (Appendix Fig. 1e) displayed identical fragment ions to the data from shark serum (Appendix Fig. 1c), which unequivocally identified the target compound as homarine. A proposed fragmentation pathway for homarine (Appendix Fig. 1f) accounts for the fragment ions observed in natural and synthetic samples of homarine (Appendix Fig. 1c, 1e).
