# Supplementary material for: Biomarkers of Whale Shark Health: A Metabolomic Approach
Source: PLoS One. 2012 Nov 15;7(11):e49379. doi: 10.1371/journal.pone.0049379 (PMC3499553; doi:10.1371/journal.pone.0049379)
Supplement: Supplementary file 2 [file pone.0049379.s002.docx]

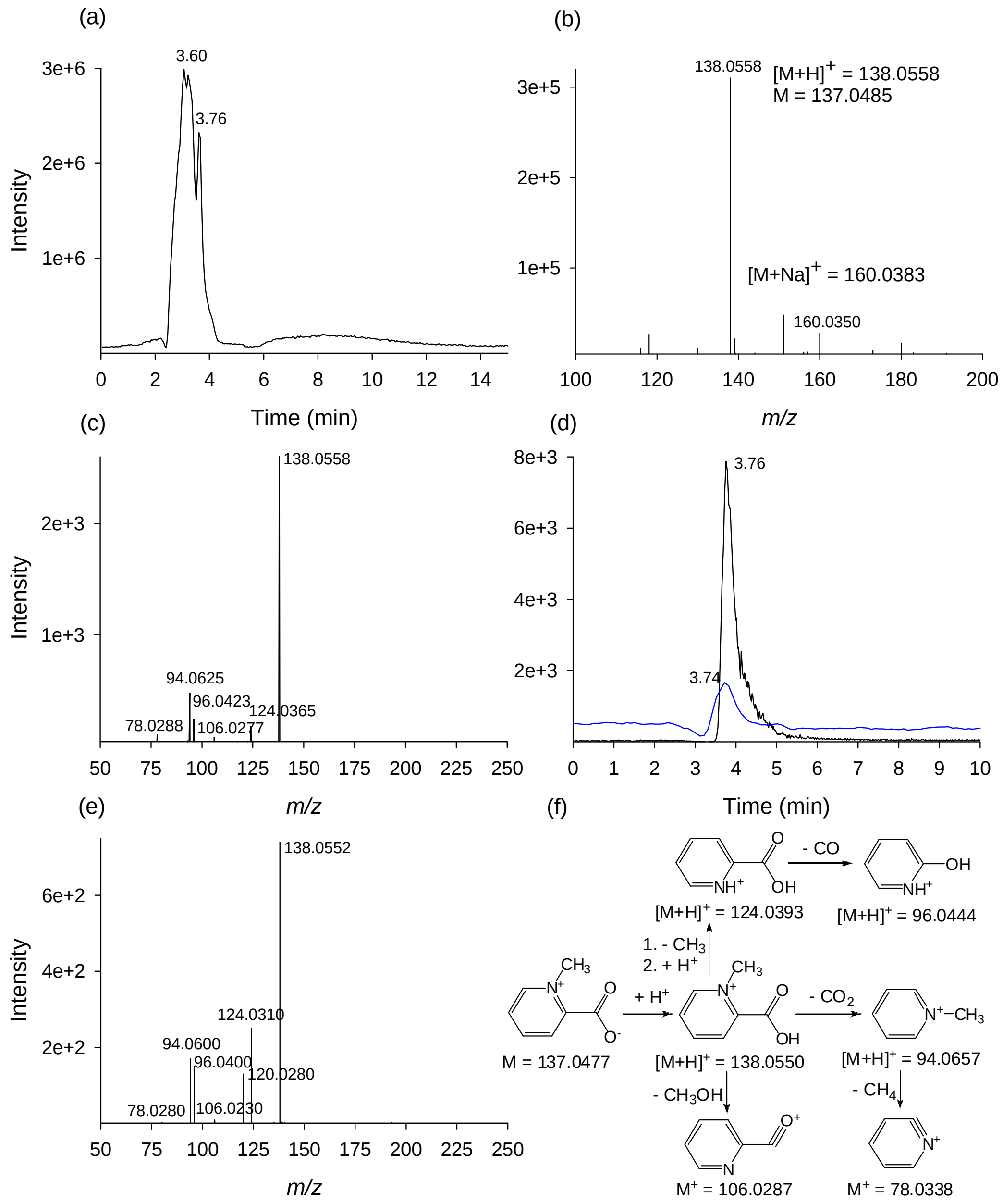

(a)
3.60
3.76
Intensity
Time (min)
(b)
138.0558
[M+H]+ = 138.0558
M = 137.0485
[M+Na]+ = 160.0383
160.0350
m/z
(c)
138.0558
94.0625
96.0423
124.0365
78.0288
106.0277
Intensity
m/z
(d)
3.76
3.74
Time (min)
(e)
138.0552
124.0310
94.0600
96.0400
120.0280
78.0280
106.0230
Intensity
m/z
(f)
- CO
[M+H]+ = 124.0393
[M+H]+ = 96.0444
1. - CH3
2. + H+
+ H+
- CO2
M = 137.0477
[M+H]+ = 138.0550
[M+H]+ = 94.0657
- CH3OH
- CH4
M+ = 106.0287
M+ = 78.0338
